# Giardiasis in the Immunocompromised: A Case Series Highlighting Diagnostic Pitfalls and Treatment Challenges

**DOI:** 10.7759/cureus.96869

**Published:** 2025-11-14

**Authors:** Alan Wang, John Greene

**Affiliations:** 1 Osteopathic Medicine, Nova Southeastern University Dr. Kiran C. Patel College of Osteopathic Medicine, Clearwater, USA; 2 Infectious Diseases, Moffitt Cancer Center, Tampa, USA

**Keywords:** giardia duodenalis, giardia intestinalis, giardia lamblia, giardiasis, gvhd, hypogammaglobulinemia, immunocompromised, metronidazole refractory giardiasis

## Abstract

*Giardia lamblia* (*G. lamblia*) is a leading cause of protozoal intestinal infection worldwide, typically presenting as a self-limiting diarrheal illness in immunocompetent hosts. In immunocompromised patients, however, giardiasis may be severe, recurrent, or refractory to standard therapy, leading to diagnostic confusion due to overlapping conditions such as chemotherapy-related diarrhea or gastrointestinal graft-versus-host disease (GVHD). We report three cases of giardiasis in patients with leukemia, each highlighting distinct clinical challenges: neutropenic fever complicated by colitis and partial bowel obstruction, recurrent refractory disease in the setting of profound hypogammaglobulinemia, and post-transplant giardiasis mimicking gastrointestinal GVHD. These cases emphasize the limitations of stool microscopy, the value of multiplex polymerase chain reaction (PCR) and immunoassays, and the increasing recognition of metronidazole treatment failure. Alternative agents such as nitazoxanide, tinidazole, or combination regimens may be required, and intravenous immunoglobulin (IVIG) can support clearance in patients with antibody deficiency. Chronic giardiasis carries significant risks of malabsorption, malnutrition, and poor oncologic outcomes, underscoring the importance of early diagnosis, tailored therapy, and supportive care in this vulnerable population.

## Introduction

Globally, *Giardia lamblia*, also known as *Giardia duodenalis* or *Giardia intestinalis,* is one of the most common causes of protozoal intestinal infection, responsible for over 280 million cases of diarrhea annually. Prevalence ranges from 2% to 5% in industrialized nations to as high as 30-40% in developing countries [1,2]. In the United States, the Centers for Disease Control and Prevention (CDC) estimates approximately 1.2 million annual cases, although most infections remain undiagnosed [1,3]. *G. lamblia* is a flagellated protozoan parasite with two distinct forms: the motile trophozoite and the environmentally resistant cyst [1]. The trophozoite, measuring approximately 15 µm, is teardrop-shaped with two anterior nuclei and multiple flagella, enabling both free swimming and attachment to the intestinal epithelium [2]. The cyst stage, in contrast, is hardy, capable of surviving for months in cold water, and resistant to routine chlorination, making waterborne transmission a major mode of spread [4].

Once ingested, cysts excyst in the duodenum, releasing trophozoites that attach to the mucosal surface. Pathogenicity arises from multiple mechanisms, including the disruption of epithelial tight junctions, impairment of brush border enzymes, and shortening of microvilli, all of which reduce absorptive capacity and contribute to maldigestion and diarrhea [1,5]. *Giardia* trophozoites also release cysteine proteases and lectins, which have cytopathic effects, further damaging epithelial integrity [5]. These changes increase intestinal permeability, alter electrolyte transport, and may trigger inflammatory responses. Host immune factors play a crucial role in clearance, particularly secretory immunoglobulin A (IgA) [4,5]; immunoglobulin deficiencies are strongly linked to persistent infections. Additionally, *Giardia* employs antigenic variation of surface proteins, known as variant-specific proteins (VSPs), to evade host immunity, thereby contributing to chronic or recurrent disease [2].

The incubation period ranges from 3 to 20 days [6]. Clinical manifestations vary widely: nearly half of infected individuals remain asymptomatic, while symptomatic patients present with watery, foul-smelling, greasy diarrhea, abdominal cramps, nausea, flatulence, and weight loss [1,3]. Chronic infection may result in prolonged diarrhea with consequences such as malabsorption and post-infectious sequelae, including irritable bowel syndrome and secondary lactose intolerance [3]. Beyond acute illness, giardiasis carries the potential for long-term health effects, which are particularly concerning in vulnerable populations, such as children and the immunocompromised [7]. Transmission occurs predominantly through ingestion of contaminated water and food, but person-to-person spread is also common, particularly in areas with poor sanitation [1]. Remarkably, the infectious dose is very low, with as few as 10 cysts capable of causing infection [3]. Zoonotic transmission from household pets and livestock has been documented [2,5], while sexual transmission, though rare, has been reported among men who have sex with men [1,3]. Beyond these traditional modes, studies have also detected *Giardia* deoxyribonucleic acid (DNA) in honey and pollen samples, suggesting an additional environmental reservoir and potential transmission pathway [8]. Outbreaks are frequently linked to daycare centers, contaminated recreational waters, and foodborne exposures, underscoring the public health significance of this pathogen.

In immunocompromised patients, especially those with hematologic malignancies, hypogammaglobulinemia, or post-transplant immunosuppression, *Giardia* infections may be more severe, recurrent, or refractory to standard therapy. Hypogammaglobulinemia, in particular, has been associated with chronic giardiasis, as IgA plays a critical role in parasite clearance. In transplant recipients, giardiasis can mimic gastrointestinal graft-versus-host disease (GVHD), complicating diagnosis and management [9]. Furthermore, treatment failures with metronidazole have been increasingly recognized, with nitroimidazole-refractory giardiasis necessitating alternative regimens such as tinidazole, nitazoxanide, or adjunctive intravenous immunoglobulin (IVIG) [5].

Given the increasing recognition of giardiasis as a significant and often underappreciated pathogen in immunocompromised populations, especially those with hematologic malignancies, we present three cases of giardiasis in patients with leukemia. These cases highlight the diagnostic challenges, recurrent disease course, and therapeutic complexities of giardiasis in vulnerable hosts.

## Case presentation

Case 1

A 57-year-old man, originally from Colombia and living in Florida since 2000, with a history of hypertension, type II diabetes mellitus, and hyperlipidemia, was admitted in August 2025 for scheduled induction chemotherapy with cytarabine, daunorubicin, and midostaurin for newly diagnosed acute myeloid leukemia (AML) identified two months earlier. His most recent trip was to New York, which was one year ago. On admission, he reported feeling generally well, with only occasional mild morning headaches. He denied fever, chills, dizziness, or rashes. Vital signs were stable: temperature 97.7°F, blood pressure 136/84 mmHg, heart rate 70 beats per minute, respiratory rate 18 breaths per minute, and oxygen saturation 95% on room air. Physical examinations and laboratory studies were unremarkable. A computed tomography (CT) scan showed right lower lobe atelectasis and right hemidiaphragm paralysis.

**Figure 1:**
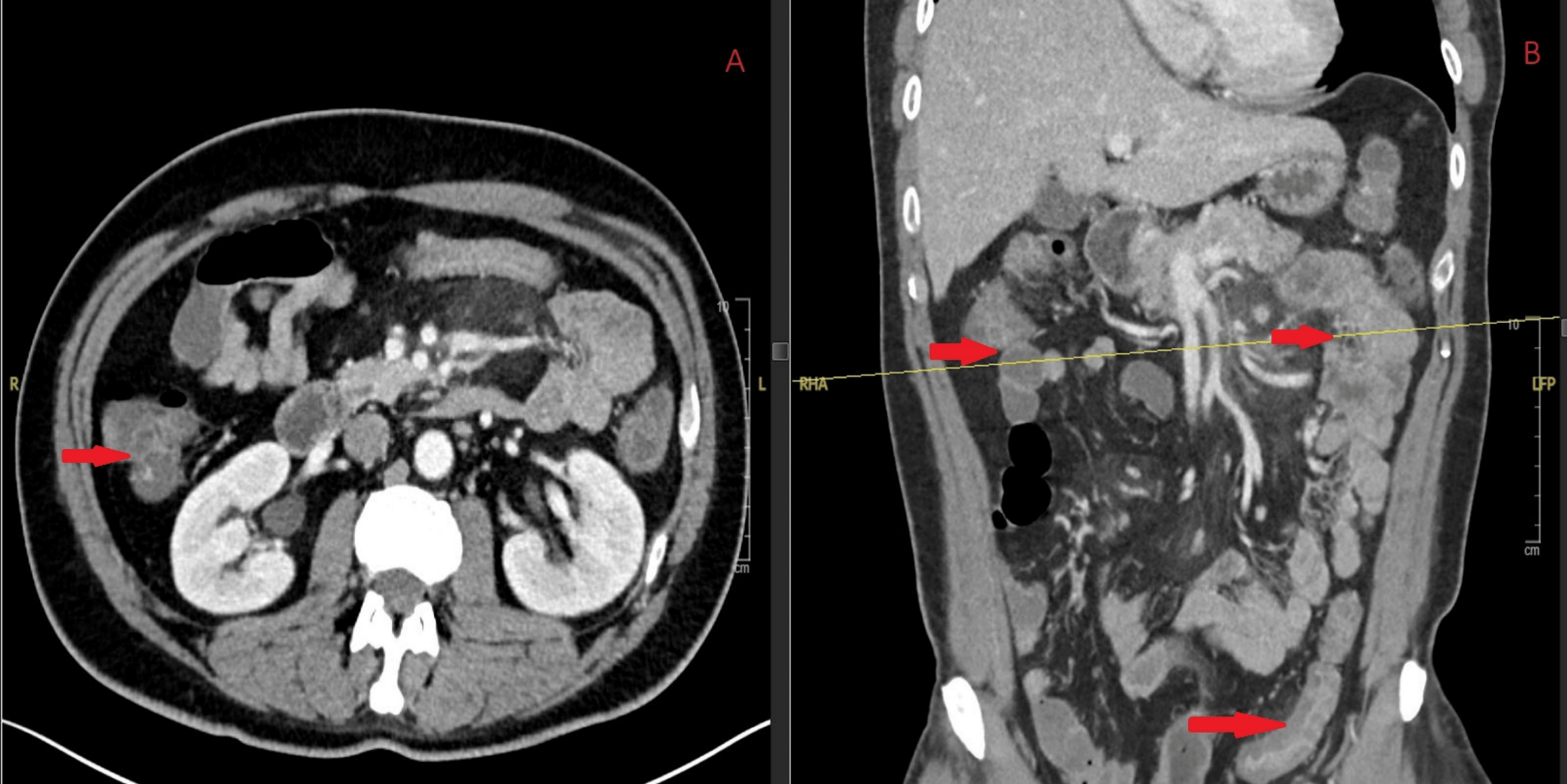
Contrast-enhanced CT of the abdomen demonstrates colonic wall thickening with pericolic fat stranding and mesenteric panniculitis (arrows). (A) Axial view. (B) Coronal view. CT: computed tomography

On hospital day 6, his absolute neutrophil count was 60 cells/µL (reference range: 1,500 to 7,700 cells/µL), and he developed a fever of 102.5°F. Blood cultures were obtained, and empiric therapy with vancomycin and cefepime was initiated. Micafungin and acyclovir were added per neutropenic fever protocol. By hospital day 9, he developed a recurrent fever of 102.7°F, and blood cultures grew *Streptococcus mitis* and *Rothia* species. Vancomycin and cefepime were continued for one week. During this time, he developed diarrhea and abdominal discomfort. A gastrointestinal pathogen panel based on multiplex polymerase chain reaction (PCR) detected *G. lamblia*, and CT imaging of the abdomen demonstrated colitis, mesenteric panniculitis, and partial bowel obstruction with a transition point at the left inguinal hernia, without evidence of pneumatosis or free air (Figure 1). He was treated with oral metronidazole 500 mg three times daily for two weeks, resulting in clearance of the infection.

Case 2

**Figure 2:**
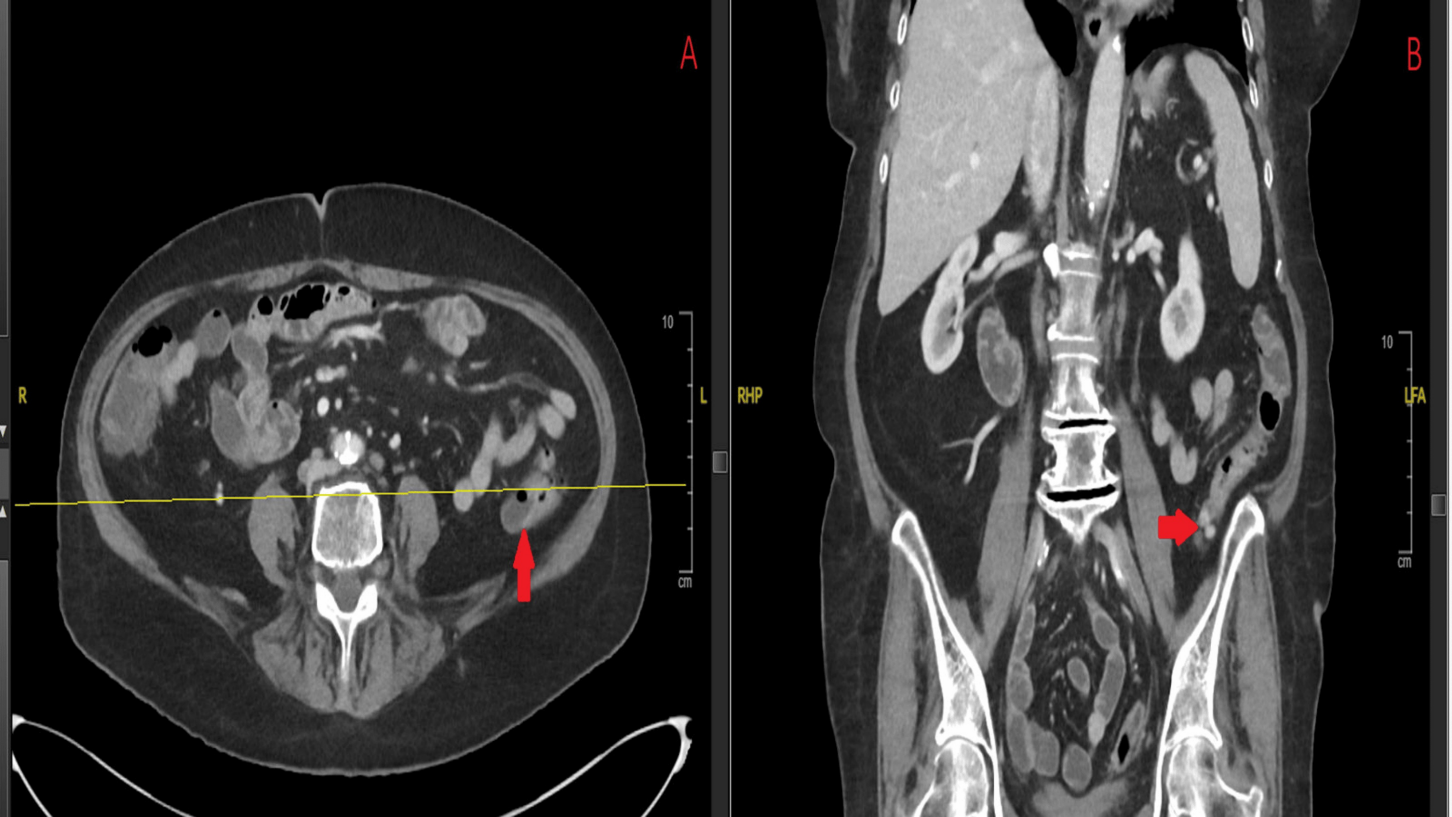
Contrast-enhanced CT of the abdomen obtained during the initial hospitalization demonstrating mucosal thickening of the left colon with pericolonic fat stranding and fluid-filled colonic loops consistent with acute diverticulitis (arrows). (A) Axial view. (B) Coronal view. CT: computed tomography

A 78-year-old woman with chronic lymphocytic leukemia (CLL) on her sixth cycle of obinutuzumab and venetoclax presented to urgent care with severe diarrhea for one week. She reported more than 10 episodes of non-bloody diarrhea per day, which initially self-resolved but recurred four days later, accompanied by mild lower abdominal cramping. She maintained adequate oral hydration throughout chemotherapy. A CT scan performed at urgent care revealed mild colonic wall thickening centered around multiple diverticula in the proximal sigmoid colon, suggestive of early diverticulitis, as well as diffuse prominent jejunal folds consistent with enteritis. She was admitted for further management. During hospitalization, the stool enzyme immunochromatographic antigen test unexpectedly detected *G. lamblia*, while *Clostridium difficile* testing was negative. Fecal lactoferrin was positive. Repeat contrast-enhanced CT of the abdomen revealed mucosal thickening of the left colon in the region of diverticulosis, consistent with acute diverticulitis. The descending colon in the left iliac fossa was diffusely fluid-filled, without evidence of bowel obstruction or perforation (Figure 2). She was started on oral metronidazole 500 mg three times daily, with symptomatic improvement. At discharge the following day, she reported decreased stool frequency (four loose bowel movements per day) and was instructed to continue metronidazole.

Two days after completing metronidazole at home, her diarrhea recurred and progressively worsened over two weeks, with up to 10 episodes per day, accompanied by abdominal pain, cramping, nausea, and vomiting. When inquiring about her social history, she was notable for having traveled out of state to Georgia three years earlier, with no recent exposure to swimming or parks. She had used exclusively bottled water until two months prior, when she briefly switched to filtered water from the refrigerator. She denied sick contacts and had a pet dog indoors. On readmission, vital signs were stable: temperature 97.9°F, blood pressure 127/57 mmHg, heart rate 69 beats per minute, respiratory rate 17 breaths per minute, and oxygen saturation 95% on room air. Laboratory testing revealed hypogammaglobulinemia (IgG 401 mg/dL, IgA 23-27 mg/dL, IgM 9 mg/dL), with details summarized in Table 1.

**Table 1:** Prominent laboratory findings in Case 2. IgG: immunoglobulin G; IgA: immunoglobulin A; IgM: immunoglobulin M

Laboratory Test	Result	Reference Range
IgG	401 mg/dL (improved to 714 mg/dL in six months)	600-1600 mg/dL (adult)
IgA	23-27 mg/dL	60-400 mg/dL (adult)
IgM	9 mg/dL	40-250 mg/dL (adult)

**Figure 3:**
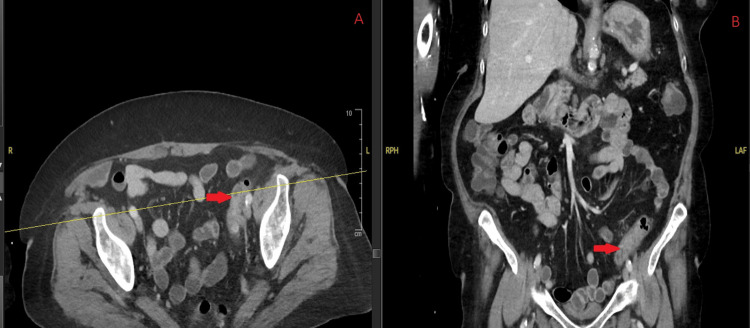
Contrast-enhanced CT of the abdomen obtained during readmission showing mild pericolic edema in the distal descending and proximal sigmoid colon consistent with uncomplicated diverticulitis (arrows). (A) Axial view. (B) Coronal view. CT: computed tomography

Stool enzyme immunochromatographic antigen test again detected *G. lamblia*; ova and parasite testing were negative. Repeat contrast-enhanced CT of the abdomen demonstrated mild pericolic edema involving the distal descending and proximal sigmoid colon, findings suggestive of mild uncomplicated diverticulitis (Figure 3). She was restarted on metronidazole 500 mg three times daily. Two days later, due to concern for treatment failure and possible resistance, her therapy was switched to nitazoxanide for refractory giardiasis. Her discharge plan included completing a three-day course of nitazoxanide, followed by a seven-day course of amoxicillin-clavulanate, for the treatment of uncomplicated diverticulitis. Monthly outpatient IVIG infusions were initiated to address hypogammaglobulinemia. An alternative discharge option included a single 2 g oral dose of tinidazole. At the one-month follow-up, the stool enzyme immunochromatographic antigen test was negative for *G. lamblia*. At six months, her IgG level improved to 714 mg/dL.

Case 3

In March 2023, a 33-year-old man with acute lymphoblastic leukemia (ALL) presented with recurrent, worsening diarrhea for three weeks. He was one year status post allogeneic hematopoietic stem cell transplant with fludarabine and total body irradiation for minimal residual disease. For prophylaxis of gastrointestinal GVHD, he had been maintained on tacrolimus, budesonide, and prednisone, in addition to acyclovir and trimethoprim-sulfamethoxazole for infectious prophylaxis.

**Figure 4:**
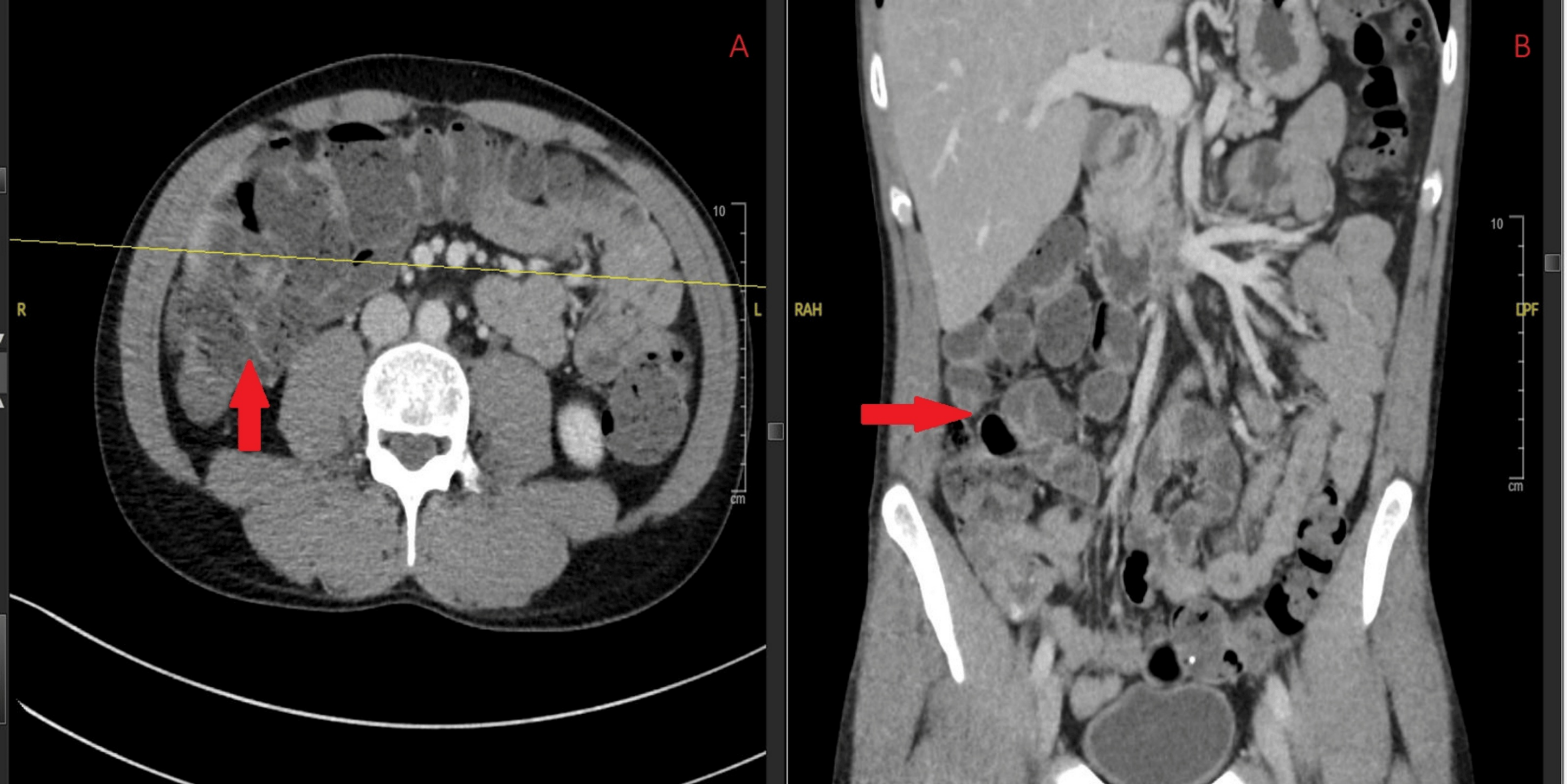
Contrast-enhanced CT of the abdomen showing right-sided colitis (arrows) with slight interval enlargement of a borderline periportal lymph node and stable subcentimeter retroperitoneal lymph nodes. (A) Axial view. (B) Coronal view. CT: computed tomography

Seven months earlier, he had been admitted with nausea, vomiting, and abdominal pain. Duodenal and gastric biopsies at that time revealed grade 1 acute GVHD, with scattered apoptosis and focal active duodenitis and gastritis. Contrast-enhanced CT of the abdomen demonstrated early right-sided colitis without obstruction, pneumatosis, or free air, along with slight interval enlargement of a borderline periportal lymph node and stable subcentimeter retroperitoneal lymph nodes (Figure 4). Concurrent stool testing via the multiplex PCR gastrointestinal panel confirmed *Giardia* enteritis.

He was treated with IVIG infusion and a 10-day course of oral metronidazole 500 mg three times daily, which he completed in October. Although his acute symptoms improved, he continued to experience intermittent diarrhea. In December 2022, tacrolimus was discontinued, though he remained on budesonide and still reported occasional cramping and loose stools without significant worsening. Despite immunosuppressive therapy for GVHD, his leukemia relapsed with 35% peripheral blasts. He subsequently received mini-hyperfractionated cyclophosphamide, vincristine, and dexamethasone (mini-HCVD) plus venetoclax. Bone marrow biopsy confirmed refractory disease with mixed-lineage leukemia (MLL) rearrangement and CD7 positivity. Around this time, he endorsed increasing fatigue and a new episode of drenching night sweats, though he denied fevers, chills, lymphadenopathy, or weight loss.

By his March 2023 visit, he again reported recurrent watery diarrhea, though he denied nausea, vomiting, or abdominal pain. Stool testing via the multiplex PCR gastrointestinal panel revealed persistent *G. lamblia*. He was treated with another seven-day course of oral metronidazole 500 mg three times daily, with subsequent resolution of symptoms.

## Discussion

Giardiasis in immunocompromised patients presents unique diagnostic and therapeutic challenges compared with the general population. While the infection is often self-limited in immunocompetent hosts, prolonged immunosuppression, impaired mucosal immunity, and antibody deficiencies contribute to persistent or relapsing infection in patients with hematologic malignancies [1,7,10,11]. In particular, corticosteroids, calcineurin inhibitors, and B-cell-depleting therapies interfere with both humoral and cellular immune responses, thereby prolonging the clinical course of the disease. Secretory IgA plays a central role in parasite clearance, and hypogammaglobulinemia has been repeatedly linked to chronic giardiasis [4,12]. This explains why patients with leukemia, transplant recipients, or those on targeted therapies such as obinutuzumab or venetoclax are especially vulnerable to recurrent infections. Compared with immunocompetent individuals, the presentation, complications, diagnostic considerations, and therapeutic outcomes of giardiasis in immunocompromised patients differ substantially. These contrasts are summarized in Table 2.

**Table 2:** Comparison of giardiasis in immunocompetent versus immunocompromised patients. GVHD: graft-versus-host disease; PCR: polymerase chain reaction; IgA: immunoglobulin A; IVIG: intravenous immunoglobulin

Feature	Immunocompetent Patients	Immunocompromised Patients
Clinical course	Often asymptomatic or self-limited; acute watery diarrhea lasting 1-3 weeks	Frequently severe, prolonged, or recurrent; may mimic GVHD or chemotherapy-related diarrhea
Complications	Usually minimal; transient malabsorption or secondary lactose intolerance	Chronic diarrhea, malabsorption, weight loss, dehydration, electrolyte imbalance, malnutrition, growth/cognitive impairment (children), and rarely death
Diagnosis	Stool ova and parasite exam (low sensitivity); antigen tests or PCR if available	Multiplex PCR or immunoassays preferred for sensitivity; repeated testing often required
Immune response	Clearance is mediated largely by secretory IgA and intact mucosal immunity	Impaired clearance with hypogammaglobulinemia, T-cell suppression, or prolonged immunosuppressive therapy
Treatment	Standard: metronidazole or tinidazole is usually effective	Higher rates of treatment failure; nitazoxanide, albendazole, or combination regimens are often required; IVIG may help in hypogammaglobulinemia
Prognosis	Excellent; most recover fully with therapy	Variable; recurrent/refractory infections are common, and nutritional decline impacts overall outcomes

Recurrent or relapsing diarrhea remains one of the most problematic features of giardiasis in this population. In oncology patients, the differential diagnosis of diarrhea is broad and often includes chemotherapy-induced toxicity, *Clostridioides difficile* infection, and gastrointestinal GVHD. These overlapping syndromes can delay testing for parasitic pathogens, particularly in non-endemic settings where clinicians may not initially consider giardiasis as a potential diagnosis. In one of our cases, gastrointestinal GVHD was strongly suspected to be based on endoscopic biopsies, yet stool testing ultimately revealed *Giardia* as the true culprit. This highlights the importance of maintaining a broad differential diagnosis in immunocompromised patients with persistent diarrhea, even in the absence of traditional epidemiologic risk factors.

Traditionally, stool microscopy has been the most common diagnostic technique for giardiasis, but its sensitivity and specificity are limited [13]. Multiple stool samples are often required to increase diagnostic yield, as cyst excretion can be intermittent. Immunodiagnostic assays, such as the enzyme-linked immunosorbent assay (ELISA), for antibody or copro-antigen detection have improved accuracy and are widely available, offering greater sensitivity than conventional microscopy [7]. More recently, molecular methods such as PCR have emerged as the most sensitive and specific approach, capable of detecting low parasite burdens and differentiating assemblages [13]. Multiplex gastrointestinal pathogen PCR panels, in particular, allow rapid and simultaneous detection of *Giardia* alongside other enteric pathogens, and should be incorporated early in the diagnostic evaluation of immunocompromised patients with unexplained or recurrent diarrhea. However, an important caveat is that *Giardia* antigen and PCR assays can remain positive for weeks to months following successful treatment, as they may detect residual antigens or non-viable DNA. Thus, these modalities are not reliable for monitoring cure, and decisions about retreatment should be guided primarily by clinical symptoms rather than persistently positive tests. This distinction is particularly important in immunocompromised patients, where over-reliance on molecular positivity may lead to unnecessary or prolonged therapy.

Treatment failure is another major obstacle in the management of giardiasis in immunocompromised hosts. Although metronidazole remains the first-line therapy, refractory disease is increasingly recognized. Proposed mechanisms for treatment failure include reduced drug activation in the parasite, encystation as a protective state, and impaired host immune clearance that allows residual organisms to persist despite therapy [1,5]. In our series, one patient developed recurrent symptoms shortly after completing an adequate course of metronidazole, ultimately requiring nitazoxanide as rescue therapy. This reflects a growing body of evidence that nitroimidazole-refractory giardiasis is not rare in immunosuppressed patients, and that alternative agents, such as tinidazole, albendazole, or nitazoxanide, may be required. In some cases, combination therapy has been suggested. In those with profound hypogammaglobulinemia, it seems reasonable to give IVIG to address the underlying deficiency and potentially aid in clearance [12].

Other potential therapeutic strategies are under investigation. Fumagillin, an antibiotic derived from *Aspergillus fumigatus*, has shown promise by inhibiting *G. lamblia* methionine aminopeptidase-2 (MetAP2), an enzyme required for the post-translational modification and activity of certain proteins critical to parasite survival [14,15]. While not yet in routine clinical use, its mechanism offers a targeted approach for refractory cases. In addition, case reports describe the use of quinacrine as salvage therapy, including in a patient with hematopoietic stem cell transplantation who achieved clearance of refractory giardiasis after failing multiple nitroimidazole courses [16]. These emerging and repurposed agents highlight the need for further research into alternative therapies for giardiasis, particularly in immunocompromised populations where standard treatments often prove ineffective.

The complications of chronic giardiasis extend beyond diarrhea and carry significant clinical consequences in individuals with compromised immunity. Prolonged infection can lead to malabsorption, secondary lactose intolerance, weight loss, dehydration, and electrolyte imbalance [7,10,17]. Over time, these effects may compound the already common malnutrition among oncology patients receiving intensive therapy. In pediatric populations, chronic giardiasis has been associated with growth retardation and cognitive impairment, reflecting its systemic impact [1,7,13]. Although less studied in adults, similar risks of cachexia and decline in functional status are likely, and mortality has been described in rare, severe cases. In our series, patients demonstrated recurrent watery diarrhea and nutritional compromise, illustrating the need for aggressive supportive care, including hydration, electrolyte management, and nutritional supplementation, in addition to antiparasitic therapy.

Management of giardiasis in immunocompromised patients, therefore, requires a multifaceted approach. Early and repeated stool testing is essential when diarrhea persists or recurs, regardless of concurrent explanations such as chemotherapy toxicity or GVHD. Clinicians should anticipate higher relapse rates and lower treatment success with metronidazole, and escalation to second-line or combination therapy should not be delayed in cases of treatment failure. Correction of underlying immune deficiencies is equally important; in the case of hypogammaglobulinemia, immunoglobulin replacement should be considered. Some transplant centers in highly endemic countries even recommend screening and treatment of *G. lamblia* infection prior to transplantation [18], reflecting its potential to complicate post-transplant outcomes. Ultimately, supportive measures, including hydration and nutritional optimization, are essential in preventing complications associated with chronic illnesses.

Our case series demonstrates several key themes relevant to clinical practice. One patient developed giardiasis during induction chemotherapy for AML, illustrating how acute immunosuppression may predispose to infection even in the absence of recent travel or high-risk exposures. Another experienced refractory giardiasis in the setting of CLL with profound hypogammaglobulinemia, highlighting the role of antibody deficiency and the need for alternative therapies. The third case, a young man post-allogeneic transplant, underscores how giardiasis can mimic GVHD and complicate transplant care. Together, these cases demonstrate varied presentations, a recurrent course, and therapeutic challenges of giardiasis in patients with hematologic malignancies.

In summary, giardiasis in immunocompromised patients is not merely a benign or self-limited infection but one that can cause substantial morbidity. Prolonged immunosuppression prolongs the disease course, recurrent diarrhea complicates diagnosis, and metronidazole-refractory disease necessitates broader therapeutic strategies. Weight loss, malnutrition, dehydration, and even death are possible outcomes if diagnosis and treatment are delayed. For clinicians, these cases underscore the importance of vigilance in evaluating diarrhea in immunocompromised patients, the early use of sensitive diagnostics, consideration of alternative therapies in refractory cases, and the provision of aggressive supportive care to mitigate complications.

## Conclusions

Giardiasis in immunocompromised patients poses significant diagnostic and therapeutic challenges. Prolonged immunosuppression, hypogammaglobulinemia, and post-transplant states predispose patients to recurrent or refractory infections, which can be mistaken for other causes of diarrhea, such as chemotherapy toxicity or gastrointestinal GVHD. Metronidazole, while the standard first-line agent, may be inadequate in this setting, necessitating consideration of alternative therapies such as nitazoxanide, tinidazole, or combination regimens, along with correction of underlying immune deficiencies. Complications, including malnutrition, electrolyte imbalance, and, in severe cases, mortality, underscore the importance of early recognition and comprehensive management. Our case series emphasizes the need for clinicians to maintain a high index of suspicion for giardiasis in immunocompromised patients with persistent diarrhea, even in non-endemic settings, and highlights the value of tailored diagnostic strategies, immune reconstitution, and timely escalation of therapy to improve outcomes.
